# Availability of COLLECT, a database for pregnancy and placental research studies worldwide

**DOI:** 10.1016/j.placenta.2017.07.014

**Published:** 2017-09

**Authors:** Leslie Myatt, James M. Roberts, Christopher W.G. Redman, Global Pregnancy Collaboration (CoLab)

**Affiliations:** a Dept of Obstetrics and Gynecology, Oregon Health & Science University, Portland, OR, USA; b Magee Women's Research Institute, Dept of Obstetrics and Gynecology and Reproductive Sciences, Epidemiology and Clinical and Translational Research, University of Pittsburgh, Pittsburgh, PA, USA; c Nuffield Dept of Obstetrics and Gynecology, University of Oxford, Oxford, UK

**Keywords:** Placenta, Preeclampsia, Database, Obstetrics, Placenta and perinatal research

## Abstract

Cost and availability of a database often impede research while the lack of compatibility inhibits collaboration by making merging of databases difficult or impossible. The Global Pregnancy Collaboration (CoLab) has promoted harmonization of studies and standardized data collection to facilitate pregnancy and placental research. Its online database, COLLECT, allows collection of minimal and optimal clinical datasets to accompany basic and applied science studies and provides a placental sample inventory system. COLLECT is available free of charge in LMIC and for $100 per month in HIC. Data is the property of the investigator but with permission can be combined into larger studies across centers and countries.

## Highlights

• High quality clinical data can usefully assist basic placental research studies.
• Designing a database is expensive and existing databases are usually incompatible.
• CoLab has designed and made available a tried and tested database at low or no cost.
• Data is the property of the investigator but can be easily combined with permission.

## 1 Introduction

The great obstetrical syndromes of preeclampsia, IUGR and preterm birth, are responsible for appreciable morbidity and mortality, however their proximate pathophysiology remains to be fully elucidated. Increasing amounts of evidence indicates that these syndromes, and preeclampsia in particular, are heterogeneous disorders with more than one subtype including those with involvement of the placenta. The burden of these disorders falls disproportionately on low and middle income countries (LMIC) where we know less of their pathophysiology and characteristics which indeed may be different from high income countries (HIC). Recent large scale studies of up to 5000 subjects have failed to accurately predict the occurrence of preeclampsia [1], [2], likely due to the heterogeneity of the condition. Hence, larger sample sizes coupled with high quality clinical and laboratory data are required to understand, predict and prevent adverse pregnancy outcomes. Logistically this is difficult to achieve in single centers but could be achieved by the merging of data from smaller studies. However, as different databases are usually incompatible this task is often difficult if not impossible. A further impediment is that design, building and operation of databases is expensive and beyond the capabilities of most investigators and particularly so in LMIC. The availability of databases capable of gathering high quality or consistent clinical data is often lacking in placental research studies, hindering generation of and combination of basic science studies.

The Global Pregnancy Collaboration (CoLab) was established with support from the Bill & Melinda Gates Foundation as an international consortium of investigators and centers to enable sharing of data and biological samples worldwide with the objective of facilitating collaborative research studies of adverse pregnancy outcomes and ultimately improve the health of women and their infants. As part of this objective the members of CoLab have published position papers which outline standards they feel are necessary for data collection, including minimal and optimal datasets [3], for placental tissue collection [4] and for harmonization of study design in relation to pre-eclampsia and related research [5]. The ultimate goal of such efforts is to allow combination of studies from diverse centers, countries and populations.

## 2 Methods

CoLab has designed and built a database, initially to facilitate preeclampsia studies worldwide. The database has been adapted by MedSciNet from that they designed for the international SCOPE study, ensuring it is tried, tested and robust. The database will be available free for use for investigators in LMIC with limited resources and available in HIC at a nominal charge ($100 per month). This is much less than that incurred for designing and operating one's own database. The database contains the minimal and optimal datasets as specified by Myatt et al., 2014 [3], facility for recording of ultrasound measurements and also contains an inventory system for sample storage and retrieval. The minimal set meets the basic requirements for a preeclampsia study and the optimal is a comprehensive dataset for in depth investigation of pathophysiology. Current developments also include a basic version of the database that can function as a birth registry. Use of the database as an adjunct to either clinical or basic placental research studies would provide a solution for those who wish to collect clinical data and ensure compatibility with other studies using the same database.

## 3 Discussion

COLLECT is an opportunity for members of the clinical and basic science placental research communities to use a high level comprehensive clinical database where none existed before. This will enable harmonization of data collection to facilitate later merging with other studies if desired and help elucidate the role of the placenta in the great obstetric syndromes [5]. The database has global application, being web-based featuring online input and viewing, with the highest standards of security and confidentiality. It is of a generic flexible structure that will support cohort and case control studies, clinical trials and can be adapted to accommodate specific research interests. As the database is of a modular design, future developments will include modules for other obstetric complications e.g. preterm birth, or study-specific parameters e.g. environmental exposures or neonatal follow-up, that each investigator can chose to add yet maintain compatibility with other components of the database. Importantly the study investigators will retain ownership of their data and it can only be shared if they give permission, with no requirement for a priori collaboration. Existing databases can be converted to this format for approx $5000 and it will be available in different languages at modest cost.

COLLECT is available now via The Data Manager of the Global Pregnancy Collaboration, email dickensonke2@mwri.magee.edu or Tel 412 641 1427.

## Sources of funding

The Global Pregnancy Collaboration is funded by the Bill & Melinda Gates Foundation (OPP1119324).
